# Genetic heterogeneity in the leader and P1-coding regions of foot-and-mouth disease virus serotypes A and O in Africa

**DOI:** 10.1007/s00705-013-1838-9

**Published:** 2013-11-13

**Authors:** M. Chitray, T. A. P. de Beer, W. Vosloo, F. F. Maree

**Affiliations:** 1 Agricultural Research Council, Onderstepoort Veterinary Institute, Transboundary Animal Diseases, Private Bag X05, Onderstepoort, Pretoria, South Africa; 2 Department of Veterinary Tropical Diseases, Faculty of Veterinary Sciences, University of Pretoria, Onderstepoort, 0110 South Africa; 3 Bioinformatics and Computational Biology Unit, University of Pretoria, Pretoria, 0002 South Africa; 4 Present Address: EMBL, European Bioinformatics Institute, Wellcome Trust Genome Campus, Hinxton, Cambridge, CB10 1SD UK; 5 Present Address: Australian Animal Health Laboratory, Geelong, Victoria Australia

## Abstract

Genetic information regarding the leader (L) and complete capsid-coding (P1) region of FMD serotype A and O viruses prevalent on the African continent is lacking. Here, we present the complete L-P1 sequences for eight serotype A and nine serotype O viruses recovered from FMDV outbreaks in East and West Africa over the last 33 years. Phylogenetic analysis of the P1 and capsid-coding regions revealed that the African isolates grouped according to serotype, and certain clusters were indicative of transboundary as well as intra-regional spread of the virus. However, similar analysis of the L region revealed random groupings of isolates from serotypes O and A. Comparisons between the phylogenetic trees derived from the structural coding regions and the L region pointed to a possibility of genetic recombination. The intertypic nucleotide and amino acid variation of all the isolates in this study supported results from previous studies where the externally located 1D was the most variable whilst the internally located 1A was the most conserved, which likely reflects the selective pressures on these proteins. Amino acids identified previously as important for FMDV structure and functioning were found to be highly conserved. The information gained from this study will contribute to the construction of structurally designed FMDV vaccines in Africa.

**Electronic supplementary material:**

The online version of this article (doi:10.1007/s00705-013-1838-9) contains supplementary material, which is available to authorized users.

## Introduction

Foot-and-mouth disease (FMD) is a highly contagious disease that affects domestic and wild cloven-hoofed animals [2, 77]. Despite all the information accumulated over the years on many aspects of FMD basic biology, there is still a lack of information regarding FMD virus transmission, maintenance, virulence and host range. Although FMD is referred to as a single disease [18], the causative agent of the disease, FMD virus (FMDV), consists of seven immunologically distinct serotypes [23, 24]. The FMDV serotypes, i.e., A, O, C, Asia 1 and the South African Territories (SAT) types 1, 2 and 3, have different global geographical distribution patterns [8–10, 18, 44, 73, 88] and are endemic in many countries. Even on the African continent, the distribution of serotypes is variable, with the SAT serotypes occurring in most regions of sub-Saharan Africa but A and O confined mostly to the central and northern parts of the region [88]. Mortality is usually low, but morbidity can reach 100 % and therefore remains a major economic concern for livestock health in many developing countries and a continued threat to disease-free countries [44]. The eradication and control of FMDV in Africa is complex and difficult due to the role of wildlife in virus spread and maintenance [82] and the presence of six of the seven serotypes, i.e., A, O, C, SAT1, SAT2 and SAT3. Serotype C has not been reported since 2004 [22].

FMDV is a non-enveloped virus containing a single-stranded RNA genome of positive polarity in the genus *Aphthovirus* of the family *Picornaviridae* [1, 2, 27]. The large open reading frame (ORF) of ~6,996 nt, which differs in length between the different serotypes [20], encodes a single polypeptide, which is co- and posttranslationally cleaved by viral proteases to give rise to the structural and non-structural proteins [3, 13, 55, 67]. Ten of the 13 cleavage events are catalysed by the virally encoded 3C protease [15, 58, 67, 78]. Translation takes place from a single open reading frame by a cap-independent mechanism at the internal ribosome entry site (IRES) [49], located in the 5’ untranslated region (UTR). There are two different sites on the RNA at which the initiation of protein synthesis occurs, resulting in the generation of two forms of L proteinase (L^pro^), Lb and the less abundant Lab, where Lb is the truncated version, which arises after the initiation of translation at the second AUG start codon [13]. Lab and Lb can cleave the L/P1 junction and ensure the proteolytic degradation of the cellular cap-binding protein complex (eIF4G), which results in the shutoff of host translation [22]. The P1 region is the viral capsid precursor and consists of the proteins 1A (VP4), 1B (VP2), 1C (VP3) and 1D (VP1). The antigenicity of the viral particles is dependent on the amino acid (aa) residues that are exposed on the surface of the capsid [56, 85]. Furthermore, it has been shown that the external capsid proteins play a role in binding to the FMDV cell-surface receptors, i.e., the RGD-dependant integrins [14, 25, 37–39, 59, 60] and heparan sulphate proteoglycans (HSPGs) [4, 36, 68].

The genetic heterogeneity of the virus, which is due to the lack of a proofreading mechanism during virus replication, has resulted in the occurrence of extensive variability as well as different lineages and antigenic variants within a serotype that have established themselves in different geographical regions [reviewed in refs. 8–10, 44, 70, 71, 75, 76, 88]. This has resulted in the need for multiple vaccine strains required for each serotype to cover the antigenic diversity when using vaccination as a control option [26]. In Africa and countries bordering Europe, the disease is mainly controlled using vaccination and restriction of animal movement. Thus, it is imperative to obtain as much information as possible regarding the FMDV prevalent on the African continent to further our knowledge on FMD epidemiology, define genetic relationships of viruses causing outbreaks [45, 47] and to enable better control strategies by successful vaccine development.

Genetic information regarding the leader (L) and complete capsid-coding (P1) region of serotype A and O viruses prevalent on the African continent is lacking, although the SAT isolates have been broadly studied in the past [8–10, 86]. For this study, the L and P1 coding regions for eight FMDV A and nine FMDV O viruses isolated between 1975 and 2003 were successfully sequenced and analysed using phylogenetic analysis, examination of sequence variability, and identification of highly conserved genomic regions relating to previously identified FMDV functional and structural biological capabilities. Non-conservative substitutions were mapped to the available O (O1BFS) [53] and A (A_10_/HOL/61) [29] capsid structures, and amino acid substitutions that may be involved in antigenic divergence were identified.

## Materials and methods

### Viruses included in this study

The sub-Saharan African isolates included in this study belong to different topotypes of FMDV serotypes A and O as defined by 1D sequencing and represent a broad geographical distribution of viruses within East and West Africa. The nine FMDV serotype O isolates and eight serotype A isolates were obtained from the Institute for Animal Health, Pirbright Laboratory, Pirbright, United Kingdom (Table 1). For the purpose of analysis, a select few complete L and P1 FMDV sequences currently available in GenBank were included (Table 1).

**Table 1:** Description of viruses used for genetic analysis of the coding sequences of the L and capsid proteins

Virus strain^a,d^	Country of origin	References	GenBank accession no.	Passage history^b^	Topotypes^c^
O/ETH/3/96*	Ethiopia	This study	EU919240	RS2	East Africa (EA)
O/UGA/5/96*	Uganda	This study	EU919247	RS2	East Africa (EA)
O/KEN/10/95*	Kenya	This study	EU919242	RS3	East Africa (EA)
O/SUD/4/80*	Sudan	This study	EU919239	RS2	East Africa (EA)
O/UGA/17/98*,▲	Uganda	This study	EU919245	RS2	East Africa (EA)
O/UGA/1/75*,▲	Uganda	This study	EU919244	RS2	East Africa (EA)
O/UGA/6/76*,▲	Uganda	This study	EU919246	RS2	East Africa (EA)
O/TAN/3/96*	Tanzania	This study	EU919241	RS2	East Africa (EA)
O/UGA/7/03*	Uganda	This study	EU919243	PK1 RS1	East Africa (EA)
O/UKG/35/2001	United Kingdom	Carrillo et al. [29]	AJ539141	-	Middle East-South Asia (ME-SA)
OFRA/1/2001	France	Nobiron et al. [61]	AJ633821	-	Middle East-South Asia (ME-SA)
O/SAR/19/2000	South Africa	Carrillo et al. [29]	AJ539140	-	Middle East-South Asia (ME-SA)
O/TAW/2/99	Taiwan	Carrillo et al. [29]	AJ539137	-	Middle East-South Asia (ME-SA)
O/TIBET/CHA/99	China	Carrillo et al. [29]	AJ539138	-	Middle East-South Asia (ME-SA)
O/CHINA/1/99	China	Zhang et al. [89]	AF506822	-	Middle East-South Asia (ME-SA)
O/SKR/2000	South Korea	Carrillo et al. [29]	AJ539139	-	Middle East-South Asia (ME-SA)
O/O10PHIL76	Philippines	Carrillo et al. [29]	AY593812	-	South East Asia (SEA)
O/O10PHIL54	Philippines	Carrillo et al. [29]	AY593811	-	South East Asia (SEA)
O/O1MANISA87	Turkey	Carrillo et al. [29]	AY593823	-	Middle East-South Asia (ME-SA)
O/AKESU/58	China	Li et al. [52]	AF511039	-	Middle East-South Asia (ME-SA)
O/11INDONESIA52	Indonesia	Carrillo et al. [29]	AY593813	-	South-East Asia
O/O1BRUGGE79	Belgium	Carrillo et al. [29]	AY593817	-	Europe-South America (Euro-SA)
O/O1ARGENTINA65	Argentina	Carrillo et al. [29]	AY593814	-	Europe-South America (Euro-SA)
O/O1CAMPOS94	Argentina	Carrillo et al. [29]	AY593819	-	Europe-South America (Euro-SA)
O/O1CAMPOS96	Brazil	Carrillo et al. [29]	AY593818	-	Europe-South America (Euro-SA)
O/O1BFS46	United Kingdom	Carrillo et al. [29]	AY593816	-	Europe-South America (Euro-SA)
O/O1BFS18	United Kingdom	Carrillo et al. [29]	AY593815	-	Europe-South America (Euro-SA)
A/CIV/4/95*,▲	Cote d’Ivoire	This study	EU919236	BTY1 RS2	Africa
A/ERI/3/98*,▲	Eritrea	This study	EU919238	BTY1 RS2	Africa
A/ETH/2/79*	Ethiopia	This study	EU919233	BTY5 RS2	Africa
A/ETH/7/92*,▲	Ethiopia	This study	EU919235	BTY1 RS2	Africa
A/NIG/4/79*,▲	Nigeria	This study	EU919234	BTY2 BHK4 RS2	Africa
A/SEN/10/97*,▲	Senegal	This study	EU919237	BTY2 RS2	Africa
A/SOM/1/78*	Somalia	This study	EU919231	BTY2 RS2	Africa
A/TAN/4/80*	Tanzania	This study	EU919232	BTY2 RS2	Africa
A/A18ZULIA40	Venezuela	Carrillo et al. [29]	AY593758	-	Europe-South America (Euro-SA)
A/A1BRAZIL75	Brazil	Carrillo et al. [29]	AY593753	-	Europe-South America (Euro-SA)
A/A17AGUARULBOS83	Brazil	Carrillo et al. [29]	AY593757	-	Europe-South America (Euro-SA)
A/APHILIPPINES50	Philippines	Carrillo et al. [29]	AY593793	-	Asia
A/A29PERU37	Peru	Carrillo et al. [29]	AY593773	-	Europe-South America (Euro-SA)
A/BRAZIL67	Brazil	Carrillo et al. [29]	AY593788	-	Europe-South America (Euro-SA)
A/A24CRUZEIRO71	Brazil	Carrillo et al. [29]	AY593768	-	Europe-South America (Euro-SA)
A/A4SPAIN62	Spain	Carrillo et al. [29]	AY593778	-	Europe-South America (Euro-SA)
A/A14SPAIN39	Spain	Carrillo et al. [29]	AY593754	-	Europe-South America (Euro-SA)
A/A5WESTERWALD73	West Germany	Carrillo et al. [29]	AY593781	-	Europe-South America (Euro-SA)
A/A5ALLIER45	France	Carrillo et al. [29]	AY593780	-	Europe-South America (Euro-SA)
A/A12VALLE119/20	Great Britain	Carrillo et al. [29]	AY593752	-	Europe-South America (Euro-SA)
A/A10HOLLAND82	Holland	Carrillo et al. [29]	AY593751	-	Europe-South America (Euro-SA)
A/A1BAYERN41	Germany	Carrillo et al. [29]	AY593759	-	Europe-South America (Euro-SA)
A/A3MECKLENBURG81	Germany	Carrillo et al. [29]	AY593776	-	Europe-South America (Euro-SA)
A/A2SPAIN7	Spain	Carrillo et al. [29]	AY593774	-	Europe-South America (Euro-SA)
A/A4WGERMANY72	West Germany	Carrillo et al. [29]	AY593779	-	Europe-South America (Euro-SA)
A/A4WGERMANY42	West Germany	Carrillo et al. [29]	AY593777	-	Europe-South America (Euro-SA)

^a^The viruses labeled “*” represent isolates from Africa

^b^RS is the number of passages on IB-RS-2 (Instituto Biologico renal suino) porcine kidney cells; BTY, on primary bovine thyroid cells; PK, on primary porcine kidney cells; and BHK, on baby hamster kidney cells. The number following the cell line indicates the number of times a virus was passaged in that particular cell line. (-) information is not available

^c^The topotypes are as described by Knowles and Samuel [18]

^d^The viruses labeled “▲” represent the African O isolates that have a codon insertion between nt 77 and 79 and the African A isolates that have a codon insertion or deletion between nt 54 and 61

### Cell culture propagation of viruses

The FMDV type O viruses were passaged for a previous study and were used directly in this study for processing, whereas the FMDV A isolates were first propagated on IB-RS-2 cells (Instituto Biologico renal suino cell line, a pig kidney cell line) to obtain a high viral titer. The IB-RS-2 cells were maintained in RPMI medium (Sigma) supplemented with 10 % foetal calf serum (FCS; Delta Bioproducts) and 1x Antibiotic-Antimycotic (100×, Gibco®), Invitrogen). Virus was added to prepared cells containing RPMI supplemented with 1 % (v/v) FCS and 1× Antibiotic-Antimycotic mixture and incubated at 37 °C until complete CPE was attained (after 48 h). Clarified cell culture supernatant containing virus was stored at −80 °C until further use.

Chinese hamster ovary (CHO) cells strain K1 (ATCC CCL-61) were maintained in Ham’s F-12 medium (Invitrogen) supplemented with 10 % FCS. Plaque assays were performed by infecting monolayer cells with the virus for 1 h, followed by the addition of a 2-ml tragacanth overlay [66] and staining with 1 % (w/v) methylene blue [54].

### RNA extraction, RT-PCR and sequencing

Total viral RNA was extracted using a modified guanidinium thiocyanate (GuSCN)-silica method [17]. The viral RNA template was reverse transcribed at 42 °C for 1 h using 10 U of AMV reverse transcriptase (Promega) and the antisense P1 primer (WDA; 5’-GAAGGGCCCAGGGTTGGACTC-3’) [12] as described previously [7]. Amplification of the L-P1 region was undertaken using the antisense P1 (WDA) primer and the sense NCR1 primer (5’-TACCAAGCGACACTCGGGATCT-3’) followed by PCR reactions using long-template Taq DNA polymerase (Roche) and thermal cycling conditions described by van Rensburg et al. [86].

PCR products of *ca.* 2,820 bp were excised from a 1 % agarose gel and purified using a Nucleospin^®^ Extract Kit (Macherey-Nagel). Purified PCR products were sequenced using a genome-walking approach with genome-specific oligonucleotides and an ABI PRISM™ BigDye® Terminator Cycle Ready Reaction Kit v3.1 (Applied Biosystems). Sequences were analysed using an ABI Prism 3100 Genetic Analyser (Applied Biosystems).

### Data analysis

Ambiguous nucleotides (nt) of the L-P1 sequences were resolved manually and assembled into a contig using the SEQUENCHER^TM^ 4.7 DNA sequence analysis software (Gene Codes Corporation, Ann Arbor, MI, USA). A consensus sequence representing the most probable nt for each position of the sequence was obtained for each isolate. Consensus sequences were translated in BioEdit 5.0.9 DNA sequence analysis software [32], and the complete L-P1 nt and aa sequences were aligned using ClustalX 1.8.1 [83]. Hypervariable regions in the complete aa alignment were defined as a linear 10-aa region containing more than 50 % variable residues. The phylogenetic analysis included the newly determined sequences as well as sequences of non-African serotype A and O isolates obtained from GenBank (Table 1). Maximum-likelihood analysis of the aligned sequences was carried out in PAUP [79] under the Aikake Information Criterion. Phylogenetic trees were constructed using the neighbour-joining (NJ), minimum-evolution (ME) and maximum-parsimony (MP) methods included in the MEGA 4.0 program [50] for the L, 1A, 1B, 1C, 1D-coding regions separately as well as the full P1-coding region. Node reliability was estimated by 1000 bootstrap replications for NJ, ME and MP trees, whilst the nucleotide substitution model of Kimura 2-parameter was employed for the NJ and ME trees and close-neighbour-interchange (CNI) with search level 1 in effect for the MP and ME trees. MEGA 4.0 [50] was utilised to determine the nt and aa variation.

Plots representing the aa variation, hydrophobicity and secondary structures for each protein were drawn using Python (http://python.org) and the matplotlib package (http://matplotlib.sourceforge.net). The number of different amino acids occurring at a specific position was used as a measure of variation, and the hydrophobicity scale of Kyte and Doolittle [51] was used to measure relative aa hydrophobicity.

The crystallographic protomers of the capsid proteins of O_1_BFS (1FOD) [53] and A_10_/HOL/61 ((1ZBE) [29] were visualized and the surface-exposed residues identified with PyMol v1.1rc2pre (DeLano Scientific LLC).

## Results

### Phylogenetic relationships and genetic heterogeneity of the serotype A and O isolates in Africa

**Fig. 1:**
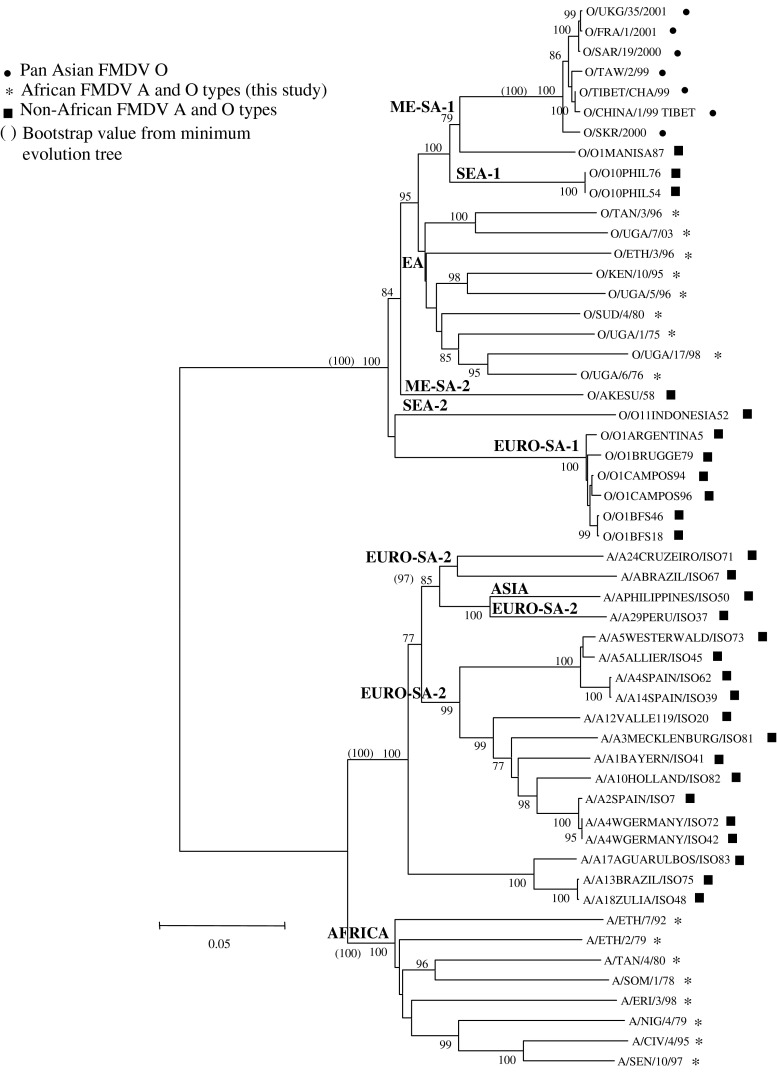
Neighbour-joining tree depicting genetic relationships for the P1 region of FMDV A and O type viruses. The Kimura 2-parameter model and bootstrap analysis (1000 replications) were applied

Phylogenetic trees based on the P1 (Fig. 1), 1B, 1C and 1D regions that included all of the A and O isolates used in this study (Table 1) revealed groupings strictly according to serotype, irrespective of the phylogenetic methodology applied. In general, analysis of the entire structural protein-coding region improved bootstrap values compared to 1D analysis alone. Phylogenetic clusters of the A and O isolates were described using the names published previously by Knowles and Samuel [44] (Table 1).

The P1 phylogeny for serotypes O viruses revealed that the African isolates clustered separately from the Pan-Asian O and non-African O isolates (Fig. 1), the latter belonging to the Middle East-South Asia (ME-SA) topotype based on 1D phylogeny [44]. The exception is O/SAR/19/2000, which was isolated in South Africa in 2000 during an outbreak caused by illegal feeding of swill to pigs [74]. This outbreak was controlled, and the virus no longer exists in southern Africa.

For the African isolates, significant bootstrap support was obtained for a group consisting of O/TAN/3/96 and O/UGA/7/03 (East African; EA-2) as well as for a group with O/KEN/10/95 and O/UGA/5/96 (East African; EA-1) and lastly for O/SUD/4/80, O/UGA/1/75, O/UGA/17/98 and O/UGA/6/76 (East African; EA-4) (Fig. 1). Furthermore, these P1 groupings were also observed when ME and MP phylogenetic models were utilised (not shown).

Clustering similar to that of the P1 region was observed for the separate gene regions, but with low bootstrap support except for 1B (O/UGA/7/03 and O/TAN/3/93, O/UGA/6/76 and O/UGA/17/98), 1C (O/UGA/17/98 and O/UGA/6/76) and 1D (OKEN/10/95 and O/UGA/5/96) groupings, which had high bootstrap support (Supplementary data, S1-S3). O/ETH/3/96 is the only representative of the EA-3 topotype in this study; thus, it did not cluster with the other isolates (Fig. 1). The nt sequence differences in the P1-coding region between members of each topotype were typically more than 15 %, similar to the cutoff defined for a topotype [47].

Globally, FMDV serotype A exists in three geographically distinct topotypes, Asia, Africa and Europe-South America (Euro-SA), based on the genetic relationships of 1D sequences [44]. Using the sequence information of the African A isolates together with P1 sequences of serotype A viruses available in the GenBank database, at least two separate clusters were observed for the type A viruses, *i.e.*, non-African and African A isolates, supported by 100 % bootstrap values for all phylogenetic methods used for the P1 (Fig. 1), 1B, 1C and 1D gene regions (Supplementary data, S1-S3). Two East African isolates, A/TAN/4/80 and A/SOM/1/78 formed a well-supported subgroup for the P1 (Fig. 1) and 1D NJ trees (Supplementary data, S3). In addition, there was a consistently strong grouping for three West African isolates, A/NIG/4/79, A/CIV/4/95 and A/SEN/1/97, in the P1 (Fig. 1), 1B, 1C and 1D NJ analyses (Supplementary data, S1-S3).

The serotype A non-African and African viruses displayed similar genetic variability when compared to serotype O. The intratypic nt sequence variation in an alignment of the 2222-nt P1-coding region for type A was calculated to be 40.4 %, whilst the corresponding region (2202-2205 nt) of type O only revealed 38.5 % variable nucleotides.

Analysis of the 1A gene region resulted in phylogenetic groupings that differed from those of the P1, 1B, 1C and 1D analyses. When performing phylogenetic analysis on the combined O and A dataset, the FMDV A and O isolates did not group strictly according to serotype (Supplementary data, S4). For example, three non-African FMDV A strains, isolated from Brazil and Venezuela (A17/AGUARULBOS/ISO83, A18/ZULIA/ISO48 and A13/BRAZIL/ISO75), grouped with O viruses from the ME-SA, SEA and EA topotypes, but with low bootstrap support. As expected, the region encoding 1A was the most conserved, exhibiting 37.9 % variant nucleotides and was the only capsid-coding region with the highest average %Ts/Tv rate of 1.0 % (Table 2). In contrast, 1D had the highest variability of 58.7 % and lowest average %Ts/Tv rate of 0.28 % (Table 2).

**Table 2:** Variation within the nucleotide and amino acid sequences of the L and P1 polyprotein in a complete alignment of the non-African and African A and O sequences

Genome region	No. of nt positions aligned^a^	No. of variant nt			% of variant nt^b^			Av. %Ts/Tv rate	No. of aa positions aligned^a^	No. of variant aa			% of variant aa^*b*^		
		All	A	O	All	A	O	All		All	A	O	All	A	O
**L**	618	318	274	267	51.5	44.3	43.0	0.470	206	111	96	61	53.9	46.6	29.6
1A	256	97	94	76	37.9	36.7	29.7	1.045	85	13	13	4	15.3	15.3	4.7
1B	657	309	253	227	47.0	38.5	34.6	0.314	218	72	53	25	33.0	24.3	11.5
1C	667	318	254	275	47.7	38.1	41.2	0.345	222	89	63	36	40.1	28.4	16.2
1D	642	377	293	277	58.7	45.6	43.0	0.283	214	116	92	64	54.2	43.0	29.9
P1	2222	1162	894	855	52.3	40.4	38.5	0.104	739	296	224	128	40.1	30.3	17.2

^a^The number of nucleotides and amino acids were based on Clustal X alignments of the complete P1-coding region of the A and O serotypes

^b^The number of variant nucleotides or amino acids for each genomic region or capsid protein relative to the total number of positions was used to estimate the percentage (%) variability

**Fig. 2:**
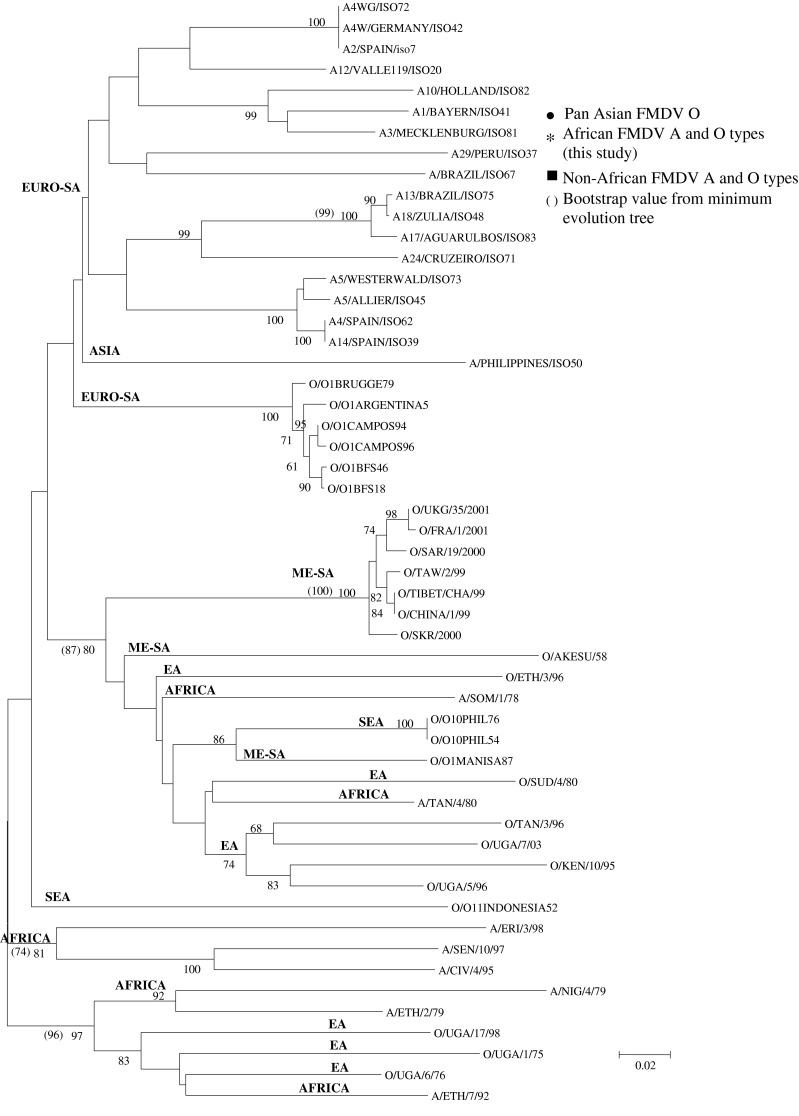
Neighbour-joining tree depicting genetic relationships for the L region of FMDV A and O type viruses. The Kimura 2-parameter model and bootstrap analysis (1000 replications) were applied

The phylogenetic trees based on the L^pro^-coding region for the combined serotype O and A dataset had similar tree topologies for the A and O isolates, independent of the phylogenetic methods employed. The NJ tree of the L^pro^-coding region (Fig. 2) showed that the viruses did not group strictly according to serotype, in contrast to those based on the structural proteins. The non-African A and O isolates that form a part of the Euro-SA lineage [44] formed separate subgroupings in the L^pro^-coding sequence NJ tree (Fig. 2). The Pan-Asian isolates formed a separate grouping with high bootstrap support (100 %). The five non-African O isolates that do not form a part of the Euro-SA lineage (isolates from the Philippines, Indonesia and China as well as the vaccine strain O1 Manisa originally isolated in Turkey; Table 1) grouped within the cluster of the African isolates, but as separate groupings. The majority of the African A and O viruses were found within two separate clusters, *i.e.*, one containing western Africa type A isolates and the other, eastern Africa type O and A (Fig. 2). Interestingly, one West African isolate, A/NIG/4/79, grouped with significantly high support with A/ETH2/79, an East African isolate. Also, a similarly strong grouping was observed for one East African (A/ERI/3/98) and two West African viruses (A/SEN/10/97 and A/CIV/4/95) (Fig. 2). There were also close relationships between African O and A viruses (O/ETH/3/96 and A/SOM/1/789; O/SUD/4/80 and A/TAN/4/80; O/UGA/6/76 and A/ETH/7/92), but these were not supported by high bootstrap values.

The nt variation for the L^pro^-coding sequence was 51.5 % compared to 52.3 % for the P1 region (Table 2). The variation in the L^pro^-coding sequence of the African isolates was further highlighted by a codon insertion between nt 77 and 79 for the Ugandan isolates O/UGA/17/98, O/UGA/1/75 and O/UGA/6/76 as well as by a codon insertion or deletion in A/CIV/4/95, A/ERI/3/98, A/ETH/2/79, A/ETH/7/92, A/NIG/4/79 and A/SEN/10/97 between nt 54 and 61 (Table 1).

### Distribution of aa variation and hypervariability of the L and P1 polypeptides

The L^pro^ aa sequence displayed significant variation for a functional protein: 46.6 % for the serotype A alignment and 29.6 % for the serotype O isolates (Table 2). At least 30.3 % (224 of 739 aa) of the aa residues were variable in the alignment of the structural proteins (translated from the P1 region) of the 26 serotype A isolates, whilst the corresponding region of the 25 serotype O isolates displayed 17.2 % (128 of 739 aa) variable residues (Table 2). As expected, the internally located 1A (15.3 % for the FMDV A and 4.7 % for FMDV O) remained the most conserved, with 1D (43.0 % for FMDV A and 29.9 % for FMDV O) the most variable FMDV capsid protein. The variability for 1C was 28.4 % for FMDV A and 16.2 % for FMDV O, compared to 24.3 % for FMDV A and 11.5 % for FMDV O when looking at 1B (Table 2).

**Fig. 3:**
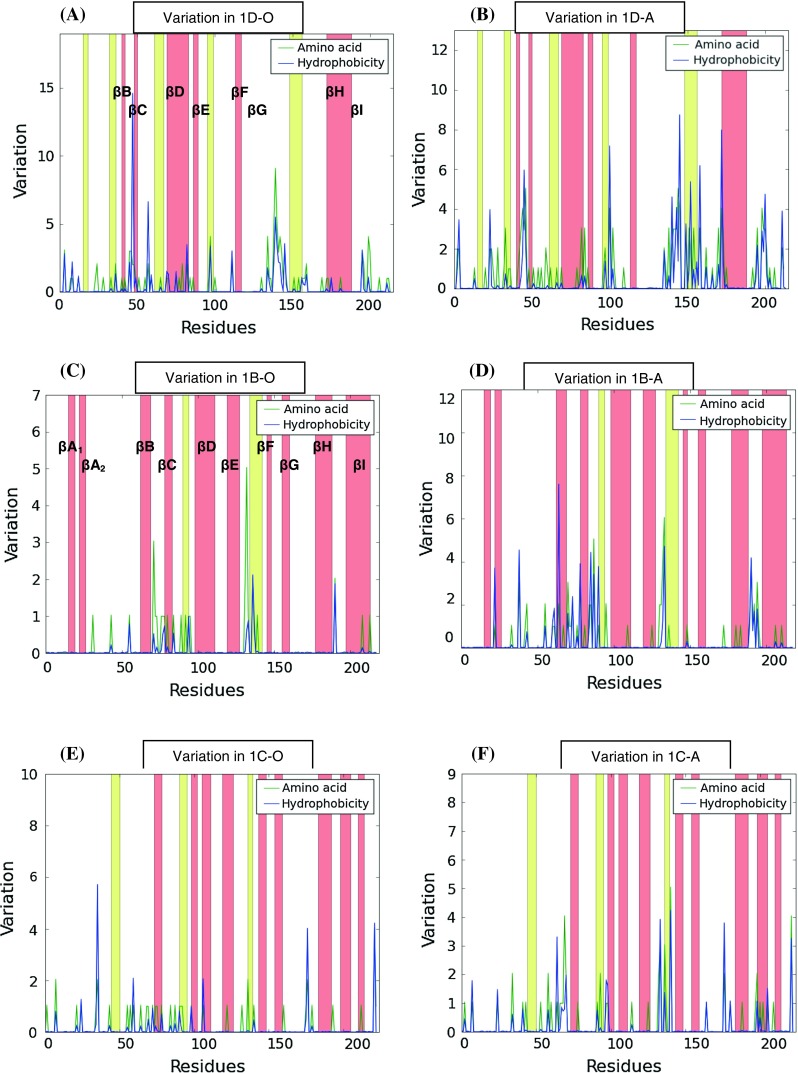
Plots representing the aa variation and hydrophobicity for 1D (**a**, **b**), 1B (**c**, **d**) and 1C (**e**, **d**) for the African type A (**b**, **d**, **f**) and O (**a**, **c**, **e**) viruses from this study. The pink bars represent the beta strands of the FMDV structure, whilst the yellow represent the alpha helices. Areas of FMDV hydrophobicity and aa variation are represented by blue and green lines, respectively. Regions of variability or hypervariable sites were defined as sites on the P1 that had five or more variable aa residues within a window of 10 residues

A systematic analysis of the capsid proteins revealed the variation not to be random but focused in local regions of hypervariability. The most variable capsid region, 1D, displayed the most regions of hypervariability. Figure 3A shows the hypervariable regions of type O at aa positions 34-60, 76-87, 135-147, 152-160, 196-213. At least seven discrete hypervariable regions (21-63, 80-87, 97-104, 135-146, 150-163, 167-176, 193-207) were identified in 1D of type A (Fig. 3B).

The conserved N-terminal motif of 1B, DKKTEETTLLEDRIL-TTRNGHTTSTTQSSVG, described by Carrillo et al. [20], was present in the African A and O sequences (results not shown). Two hypervariable sites, residues 72-85 within the βB-βC loop and 131-141 in the βE-βF loop, were mapped within 1B of type O (Fig. 3C). 1B of type A displayed the same two hypervariable regions, residues 61-92 and 129-139, and a third hypervariable region, 188-198 (βH-βI loop; Fig. 3D).

Most of the 1C aa substitutions for type O were concentrated in one hypervariable region, i.e. 68-80. A second region with significant variability worth mentioning was residues 175-181, where three residue positions displayed high entropy and were located within a surface-exposed loop of 1C (Fig. 3E). The latter was situated in the β-β ‘knob’ of 1C and included the epitope site 4 for serotype O [43]. At least three hypervariable regions were identified in the type A alignment, i.e. residues 58-72, 132-142 and 197-211 (Fig. 3F).

The 1A protein of serotype O was most conserved, with only four variable residues and hypervariable regions that were not common for 1A (not shown).

The amino acids that have previously been identified as critical for FMDV were compared to the complete aa sequence alignment of the African and non-African A and O isolates from this study and are summarized in Supplementary data S5, showing that the aa residues important for FMDV function are conserved.

### Investigation of possible heparan sulphate usage for O/KEN/10/95

Amino acid sequence alignments of all the African FMDV A and O viruses investigated in this study revealed an Arg at position 56 of 1C for only one African virus, O/KEN/10/95 (results not shown). The studies of Sa-Carvalho et al. [68] and Fry et al. [28] confirmed the importance of the R56 residue of 1C for HS binding and cell culture adaptation. FMDV plaque assays in CHO-K1 cells (Table 3) confirmed that O/KEN/10/95 was the only virus that was able to infect and replicate in this cell line. Taking all of the serotype O capsid-sequence data together, 25 of the 27 O isolates had a His residue at position 56 of 1C, and they might therefore require integrins to replicate in cell culture.

**Table 3:** Amino acid variation at amino acid position 56 of 1C and the relative infectivity of African O viruses in CHO-K1 cells

African O viruses	1C residue 56^a^	Titer in CHO-K1 cells (pfu/ml)^2^	Plaque size (in mm)^b^
O/UGA/5/96	His	-	-
O/SUD/4/80	His	-	-
O/ETH/3/96	His	-	-
O/TAN/3/96	His	-	-
O/KEN/10/95	Arg	3.63 × 10^4^	<2
O/UGA/7/03	His	-	-
O/UGA/1/75	His	-	-
O/UGA/17/98	His	-	-
O/UGA/6/76	His	-	-

^a^His=histidine; Arg=arginine

^b^(-) No growth was observed in CHO-K1 cells

### Amino acid sequence variation in relation to structure

**Fig. 4:**
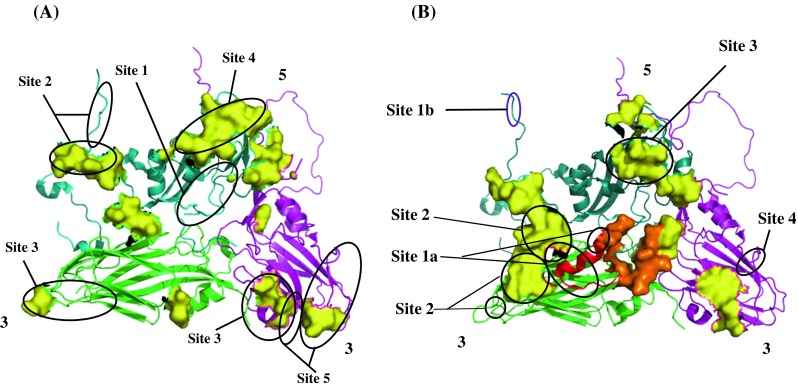
The location of surface-exposed amino acid differences in the capsid proteins of African serotype A (**A**) and O (**B**) viruses on the crystallographic protomers of O1BFS (1FOD; Logan et al. [53]) and A_10_/61 (1QQP; Fry et al. [28]). The protein subunits are colour-coded: 1D (teal), 1B (green), 1C (magenta) and the G-H loop of 1D (red). The G-H loop of A_10_/61 (residues 138-158) is not resolved in the available structure and is not indicated here (**A**). The hypervariable amino acid positions in the G-H loop of O1BFS are shown in orange (**B**). 1A has been hidden from the structure. The fivefold and threefold axes of the capsid are indicated. The positions of surface-exposed residues with high variability are indicated in yellow. The surface view was made in Pymol

Vaccines based on A22/Iraq/64, A/ERI/98 and O1Manisa are recommended for the control of FMD in Africa [33]. We examined the variation within the deduced amino acid sequences of the capsid proteins of the African O and A isolates and compared the surface-exposed regions with those of the three recommended vaccine strains. Regions with high aa variability in an alignment of the capsid proteins were mapped onto the X-ray crystallographic structures of type A (A_10_/HOL/61; 1QQP) [29] and O (O_1_BFS; 1FOD) [53] viruses. Figure 4 shows that the regions of variability were mostly located on surface-exposed regions of the virion. Not all of the aa side chains within a variable region were exposed on the surface. Closer inspection of each aa position within a region of hypervariability indicated that positions with high variability had side chains exposed to the microenvironment of the virion.

For serotype A viruses, most of the hypervariable regions outside the 1D βG-βH loop were concentrated around the 5-fold and 3-fold axes of the virion and the C-terminus of 1D (Fig. 4) and correlated to a large extent with residues previously found to be involved in escape from neutralization by monoclonal antibodies (Table 4). Furthermore, many of the putative epitopes were probably discontinuous. For example, there was close proximity of 1B residue 2191 and 1C residues 3068-3071 and 3197-3198 around the 3-fold pore of the virion (Fig. 4). Similarly the regions of variability for type O correlated strongly with epitopes previously identified with distribution around the 5-fold and 3-fold axes of the virion (Fig. 4; Table 4).

**Table 4:** Comparison of hypervariable regions identified in this study and previously identified neutralizing sites of type A and O FMDV

FMDV	Axis	Capsid	Β-sheet structure	Hypervariable region indentified in this study^a,c^	Previously identified neutralisation sites^b,c^
Type O	5×	1D	B-C	1034-1060	Site 3: 1043-1048 T-help: 1024-1042
	2×	1D	G-H	1135-1147	Site 1a: 1144-149;1154
		1B	E-F	2131-2141	Site 2: 2131-2134; 2188
	3×	1B	B-C	2072-2085	Site 2: 2070-2078
		1C	B-C		Site 4: 3056-3058
		1D	C_T_	1196-1213	Site 1b: 1206-1208
Type A	5×	1D	H-I	1167-1176	Site 4: 1169; 1175-1178
	2×	1D	G-H	1135-1146 & 1150-1163	Site 1: 1142-1157; 1138-1144
		1B	H-I	2188-2198	Site 3: 2196
	3×	1B			Site 3: 2079; 2082-2088
		1C	B-B	3058-3072	Site 5: 3058-3061
		1C	B-C	3058-3072	Site 5: 3069-3070
		1D	C_T_	1193-1207	Site 2: 1198; 1200-1212
		1C	E-F	3132-3142	Site 3: 3136-3139

^a^The hypervariable regions were derived from the alignment of serotype A and O capsid sequences

^b^The antigenic sites are a summary of those described by Thomas et al. [80]; Baxt et al. [11]; Bolwell et al. [16]; Saiz et al. [72] (type A); Kitson et al. [43]; Crowther et al. [21]; Barnett et al. [5]; Barnett et al. [6] (type O)

^c^The amino acid residues have been numbered independently for each FMDV protein. For each residue, the first digit indicates the protein (1D, 1B or 1C), and the last three digits indicate the amino acid position

## Discussion

The data from the analysis of the complete capsid-coding region, P1, as well as the individual capsid-coding regions indicated that very similar tree topologies existed for the different genomic regions when comparing the African A and O viruses with those from other regions of the world. In general, analysis of the entire structural protein-coding region improved bootstrap values relative to 1D analysis alone. The longer the capsid-coding region included in the analysis, the more accurate the relationship conclusion. This supports the view that sequencing of the entire capsid-coding region, rather than 1D alone, is desirable in molecular evolution studies.

Phylogeny based on the NJ trees of the P1, 1B, 1C and 1D sequences resulted in the grouping of viruses according to serotype. In addition, the A and O virus clusters could be further divided into separate groupings of the African and non-African A and O isolates, which were observed for the P1, 1B, 1C and 1D NJ, ME and MP trees.

The separate groupings of the African and non-African A viruses support previous findings for type A viruses. These could be grouped into three major restricted genotypes, i.e., Euro-South America, Asia and Africa, based on 1D phylogeny (this study only included FMDV A viruses from Euro-South America and Africa) [44, 46, 57].

Similarly, based on 1D phylogeny, type O viruses were divided into three groups: those originating from Asia, Europe-South America and the Far East [44, 69, 73, 74]. The P1 phylogeny therefore supports the three major virus groups within serotype O. The eastern and western African O viruses were grouped together with the SEA and ME-SA lineages, together with the Pan-Asia strain [44, 45, 73], albeit as lineages restricted to geographic regions (East Africa-1, 2, 3, 4 and West Africa). Furthermore, the phylogeny is indicative of the transboundary spread of FMDV in Africa among the East African countries, Uganda, Kenya, Somalia and Tanzania, that are in close proximity to each other, which is also true for the West African countries, i.e. Nigeria, Ivory Coast and Senegal. The groupings also indicated that the East African and West African viruses fall into separate large groups. Another well-supported grouping was observed for the P1, 1B and 1C trees (all methodologies) for O/UGA/1/75, O/UGA/6/76 and O/UGA/17/98, with a maximum of 15.1 % nt and 6.5 % aa substitutions in any pairwise alignment. This grouping most likely signifies that the 1998 outbreak strains re-emerged from older strains that have been maintained in the endemic area since the early 1970s, i.e. from 1975 to 1998 (23 years).

There was a difference in the groupings for the 1A trees when compared to the P1 and other capsid-coding gene regions where three non-African A isolates clustered with the non-African O viruses (for all phylogenetic methodologies). The phylogenetic tree representing the region encoding the L protein differed from that of the structural proteins where sub-grouping according to serotype was much less apparent, which was consistent with previous findings for this region [81, 86].

Interestingly, certain A and O African viruses clustered together and also did not separate into geographical regions such as East and West Africa as observed for the structural coding regions. For example, bootstrap support of 73 % for the L-region NJ tree was observed for the grouping of O/UGA/17/98, O/UGA/1/75, O/UGA/6/76 & A/ETH/7/92, which was not observed with the 1A, 1B, 1C, 1D and P1 phylogenetic analysis. This suggests that the African viruses share similarities or are closely related when comparing the L sequences, irrespective of serotype. Taking into account the extensive, uncontrolled movement of animals across the borders and the ease of virus spread and infection of multiple serotypes in one animal, the role of recombination events in the genetic diversification of FMDV cannot be excluded. Although we did not perform a study on the occurrence of recombination, the similarities present between FMDV A and O L sequences could be due to the occurrence of intertypic recombination events [30, 40–42, 86].

Due to the high mutation rates of FMDV, it is likely that even brief epidemics might result in the generation of substantial antigenic variability [35]. However, the adaptive significance of this variation remains unclear [34]. The antigenicity of FMDV is attributed to the aa residues that are exposed on the surface of the capsid [56]. An important immunogenic determinant, the 1D G-H loop [3], exhibited a high degree of variation for the A and O isolates included in this study. Consequently, aa changes in this region are most likely involved in the appearance of novel antigenic types. Analyses of antigenic sites of picornaviruses have been carried out using neutralising monoclonal antibodies (Mabs) to select and screen Mab-resistant mutants. Sequence analysis of these mutants resulted in the identification of five antigenic sites of serotype O virus, i.e., O_1_ Kaufbeuren [21, 44], and six sites for the FMDV A viruses [44]. Alignments of the aa sequences of the African A and O viruses indicated that the regions of variability identified corresponded to the known antigenic sites, which points to the fact that the location of antigenic sites are structurally conserved for the African A and O viruses. In addition to these sites, other regions of variability were identified for both the FMDV O and A African isolates from the aa variability plots. These regions could potentially be antigenic determinants, which may be difficult to map by the classical methodology of MAb-resistant escape mutants. We have recently shown that an approach combining sequence variation with structural data and antigenic variation results in the reasonably accurate identification of novel antigenic determinants on the virion surface [65].

The aligned L^pro^ aa sequences displayed marked variation in both the Lab and Lb regions (not shown); however, despite this variation, the aa residues identified as being critical for the L^pro^ function were highly conserved, i.e., the residues C53, H153 and D168 required for L^pro^ catalytic activity, the E81 residue required for L^pro^ autocatalysis, and two His residues (H114 and H143) important for cleavage of the translation initiation factor, eIF4G, [31, 48, 62, 63]. A comparison of the L/P1 cleavage sequence at the C-terminus of the L protein and N-terminus of the 1A protein of the FMDV non-African A types revealed a sequence of R(Q/W)KLK*GAGQ (* indicates cleaved peptide bond), whereas the African A types included in this study had the sequence K(R)R(K)LK*GAGQ (results not shown). Both the FMDV non-African and African O types revealed a sequence of (K/R)(K/R)L(K/R)*GAGQ (* indicates cleaved peptide bond) (results not shown). These observations compared well with the L^pro^/1A junction previously described for serotypes A, O and C [76], where the residues K(R)R(K)LK(R) at the L^pro^ C terminus and the GAGQ at the 1A N terminus were observed. These results suggest that for all the A and O types included in this study, the conserved sequence XXLK(R)*GAGQ (where X is either K or R) is sufficient for L/P1 cleavage by L^pro^.

The degree of hydrophobicity/hydrophilicity of the loops connecting the β chains varied between the African A and O surface proteins. Hydrophilic β-β loops tend to be exposed on the protein surface, sometimes protruding from the protein core, and are candidates for antibody binding [87]. Overall, the aa sequence variation observed for the FMDV A and O viruses included in this study showed that the A viruses exhibited more variation, possibly indicating that the A viruses evolved rapidly, which supports studies by Bachrach [2] and Brooksby [19]. Additionally, Tully and Fares [84] showed that among all of the FMDV serotypes, serotype A is the most divergent and that adaptive evolution has occurred in the 3C protease (involved in RNA replication and processing of the polyprotein) and 2B (involved in membrane rearrangements), which supports the hypothesis of selection for faster replication in serotype A.

Neff et al. [59] showed that a variant of the type O1 virus containing an Arg at residue 56 of 1C required only HS binding to replicate in CHO-K1 cells but that another variant with a His residue at this position required integrins to replicate in cell culture. Interestingly, in this study, it was shown that O/KEN/10/95 was the only African virus to have this Arg residue at residue 56 of 1C, and it was indeed able to replicate in CHO-K1 cells. However this virus has been passaged three times on IB-RS-2 cells, and it is possible that the mutation arose during cell culture passage. Additionally, various aa residues that were previously identified as important for playing a role in various functions for FMDV were found to be conserved for the A and O isolates (see “Results”).

It is clear from the outbreaks of FMD during the last two decades that there is a continuing threat to the livestock industry. The results presented here show distinct geographical grouping of serotype A and O viruses in Africa, although common ancestry with the Euro-South American-Asian topotypes is clear. The natural diversification of FMDV occurs during replication in infected animals and results in the rapid generation of mutants and the ability to persist and to spread amongst livestock. Thus, continuous surveillance and an active molecular epidemiology program increases our knowledge with regard to FMDV phylogenetic relationships, virus antigenicity, and the ability of existing vaccine strains to provide protection against emerging and re-emerging viruses.

## Electronic supplementary material

Below is the link to the electronic supplementary material.

Supplementary material 1 (DOCX 488 kb)
