# Supplementary material for: Genetic heterogeneity in the leader and P1-coding regions of foot-and-mouth disease virus serotypes A and O in Africa
Source: Arch Virol. 2013 Nov 13;159(5):947–61. doi: 10.1007/s00705-013-1838-9 (PMC4010724; doi:10.1007/s00705-013-1838-9)
Supplement: Supplementary file 1 — Supplementary material 1 (DOCX 488 kb) [file 705_2013_1838_MOESM1_ESM.docx]

**S1**: Neighbour-joining tree depicting gene relationships for the 1B region of A and O type viruses. Kimura-2 parameter and bootstrap (1000 replications) were applied.

O/O1CAMPOS96

O/O1BFS18

O/O1CAMPOS94

O/O1BFS46

O/O1ARGENTINA5

O/O1BRUGGE79

O/O11INDONESIA52

O/AKESU/58

O/UGA/7/03

O/TAN/3/96

O/UGA/5/96

O/ETH/3/96

O/UGA/6/76

O/UGA/17/98

O/UGA/1/75

O/KEN/10/95

O/SUD/4/80

O/O10PHIL54

O/O10PHIL76

O/O1MANISA87

O/FRA/1/2001

O/UKG/35/2001

O/SAR/19/2000

O/SKR/2000

O/TAW/2/99

O/CHINA/1/99

O/TIBET/CHA/99

A/A2SPAIN/ISO7

A/A4WGERMANY/ISO42

A/A4WG/ISO72

A/A10HOLLAND/ISO82

A/A1BAYERN/ISO41

A/A3MECKLENBURG/ISO81

A/A12VALLE119/ISO20

A/A14SPAIN/ISO39

A/A4SPAIN/ISO62

A/A5ALLIER/ISO45

A/A5WESTERWALD/ISO73

A/ABRAZIL/ISO67

A/APHILIPPINES/ISO50

A/A24CRUZEIRO/ISO71

A/A29PERU/ISO37

A/A17AGUARULBOS/ISO83

A/A13BRAZIL/ISO75

A/A18ZULIA/ISO48

A/ETH/7/92

A/TAN/4/80

A/SOM/1/78

A/ETH/2/79

A/ERI/3/98

A/NIG/4/79

A/CIV/4/95

A/SEN/10/97

100

100

100

100

100

92

75

74

98

81

78

82

79

73

100

100

100

95

91

72

100

100

0.05

(100)

(71)

(86)

(100)

*

*

*

*

*

*

*

*

*

*

*

*

*

*

*

*

*

•

*

Pan Asian FMDV O

African FMDV A and O types (this study)

Non-African FMDV A and O types

( )

Bootstrap value from minimum

Evolution tree

•

•

•

•

•

•

•

**S2**: Neighbour-joining tree depicting gene relationships for the 1C region of A and O type viruses. Kimura-2 parameter and bootstrap (1000 replications) were applied.

O/O1CAMPOS94

O/O1CAMPOS96

O/O1BRUGGE79

O/O1BFS46

O/O1BFS18

O/O1ARGENTINA5

O/O1CASEROS35

O/AKESU/58

O/TAN/3/96

O/UGA/7/03

O/SUD/4/80

O/UGA/1/75

O/UGA/17/98

O/UGA/6/76

O/ETH/3/96

O/KEN/10/95

O/UGA/5/96

O/O10PHIL76

O/O10PHIL54

O/SKR/2000

O/UKG/35/2001

O/FRA/1/2001

O/SAR/19/2000

O/TAW/2/99

O/TIBET/CHA/99

O/CHINA/1/99

O/O11INDONESIA52

A/A18ZULIA/ISO48

A/A13BRAZIL/ISO75

A/A17AGUARULBOS/ISO83

A/A24CRUZEIRO/ISO71

A/A29PERU/ISO37

A/APHILIPPINES/ISO50

A/ABRAZIL/ISO67

A/A4SPAIN/ISO62

A/A14SPAIN/ISO39

A/A5ALLIER/ISO45

A/A5WESTERWALD/ISO73

A/A1BAYERN/ISO41

A/A3MECKLENBURG/ISO81

A/A12VALLE119/ISO20

A/A10HOLLAND/ISO82

A/A4WGERMANY/ISO42

A/A4WG/ISO72

A/A2SPAIN/ISO7

A/TAN/4/80

A/SOM/1/78

A/ERI/3/98

A/ETH/2/79

A/ETH/7/92

A/NIG/4/79

A/CIV/4/95

A/SEN/10/97

100

99

78

100

100

100

94

97

98

83

79

99

98

100

98

100

90

91

98

100

99

100

73

0.05

(100)

(100)

(99)

•

*

Pan Asian FMDV O

African FMDV A and O types (this study)

Non-African FMDV A and O types

( )

Bootstrap value from minimum

Evolution tree

*

*

*

*

*

*

*

*

*

*

*

*

*

*

*

*

*

•

•

•

•

•

•

•

**S3**: Neighbour-joining tree depicting gene relationships for the 1D region of A and O type viruses. Kimura-2 parameter and bootstrap (1000 replications) were applied.

O/UKG/35/2001

O/FRA/1/2001

O/SAR/19/2000

O/TAW/2/99

O/TIBET/CHA/99

O/CHINA/1/99

O/SKR/2000

O/O10PHIL76

O/O10PHIL54

O/O1MANISA87

O/TAN/3/96

O/UGA/7/03

O/KEN/10/95

O/UGA/5/96

O/UGA/17/98

O/SUD/4/80

O/UGA/1/75

O/UGA/6/76

O/ETH/3/96

O/AKESU/58

O/O11INDONESIA52

O/O1BRUGGE79

O/O1ARGENTINA5

O/O1CAMPOS94

O/O1CAMPOS96

O/O1BFS46

O/O1BFS18

A/A24CRUZEIRO/ISO71

A/ABRAZIL/ISO67

A/A29PERU/ISO37

A/APHILIPPINES/ISO50

A/A4SPAIN/ISO62

A/A14SPAIN/ISO39

A/A5WESTERWALD/ISO73

A/A5ALLIER/ISO45

A/A12VALLE119/ISO20

A/A10HOLLAND/ISO82

A/A1BAYERN/ISO41

A/A3MECKLENBURG/ISO81

A/A2SPAIN/ISO7

A/A4WG/ISO72

A/A4WGERMANY/ISO42

A/A17AGUARULBOS/ISO83

A/A13BRAZIL/ISO75

A/A18ZULIA/ISO48

A/ETH/2/79

A/SOM/1/78

A/TAN/4/80

A/ETH/7/92

A/ERI/3/98

A/NIG/4/79

A/SEN/10/97

A/CIV/4/95

99

99

100

100

76

96

92

100

73

93

85

76

100

99

100

100

100

99

96

83

77

99

91

79

94

99

69

0.05

(98)

(100)

•

•

•

•

•

•

•

*

*

*

*

*

*

*

*

*

*

*

*

*

*

*

*

*

•

*

Pan Asian FMDV O

African FMDV A and O types (this study)

Non-African FMDV A and O types

( )

Bootstrap value from minimum

Evolution tree

**S4**: Neighbour-joining tree depicting gene relationships for the 1A region of A and O type viruses. Kimura-2 parameter and bootstrap (1000 replications) were applied.

O/UKG/35/2001

O/FRA/1/2001

O/SAR/19/2000

O/TAW/2/99

O/TIBET/CHA/99

O/CHINA/1/99

O/SKR/2000

O/O1MANISA87

O/O10PHIL76

O/O10PHIL54

O/TAN/3/96

O/UGA/7/03

O/SUD/4/80

O/ETH/3/96

O/KEN/10/95

O/UGA/5/96

O/UGA/1/75

O/UGA/17/98

O/UGA/6/76

O/AKESU/58

A17/AGUARULBOS/ISO83

A18/ZULIA/ISO48

A13/BRAZIL/ISO75

O/O11INDONESIA52

O/O1ARGENTINA5

O/O1CAMPOS96

O/O1BFS46

O/O1BRUGGE79

O/O1CAMPOS94

O/O1BFS18

A2/SPAIN/iso7

A4WG/ISO72

A4W/GERMANY/ISO42

A1/BAYERN/ISO41

A10/HOLLAND/ISO82

A3/MECKLENBURG/ISO81

A12/VALLE119/ISO20

A24/CRUZEIRO/ISO71

A5/ALLIER/ISO45

A5/WESTERWALD/ISO73

A14/SPAIN/ISO39

A4/SPAIN/ISO62

A/BRAZIL/ISO67

A/SOM/1/78

A/PHILIPPINES/ISO50

A29/PERU/ISO37

A/NIG/4/79

A/ETH/2/79

A/TAN/4/80

A/ETH/7/92

A/ERI/3/98

A/SEN/10/97

A/CIV/4/95

91

75

100

75

81

99

100

99

88

100

96

99

73

99

0.02

(97)

(99)

•

*

Pan Asian FMDV O

African FMDV A and O types (this study)

Non-African FMDV A and O types

( )

Bootstrap value from minimum

Evolution tree

•

•

•

•

•

•

•

*

*

*

*

*

*

*

*

*

*

*

*

*

*

*

*

*

**S5**: A comparison of previously identified critical residues for FMDV to the FMDV A and O types from this study

| **aa Regions identified as important** | **Protein: aa residue/s *** | **A (African and non-African viruses): N (number of isolates aligned) = 26** | **A (African viruses): N=8** | **O (African and non-African viruses): N=27** | **O (African viruses): N=9** |
| --- | --- | --- | --- | --- | --- |
| N-terminal myristylation site & swine and bovine T-cell epitope (Blanco *et al*., 2001) | **1A**: 19 - 35 | *c* | *c* | Y31H (1) | Y31H (1) |
| A Q residue distinguishes the SAT types from the Euro-Asiatic lineages (Carrillio *et al*., 2005). | **1A**: 73 | S (25)  N(1) | S (8) | S (27) | S (9) |
| The I residue is potentially specific for the SAT2 and SAT3 viruses (Carrillo *et al*., 2005) | **1A**: 76 | F (26) | F (8) | F (27) | F (9) |
| A valine is potentially specific for SAT1 (Carrillo *et al*., 2005). | **1A**: 80 | F (26) | F (8) | F (27) | F (9) |
| Identified 1B T-cell epitopes (Perez *et al*., 2000). | **1B**: 133 – 153  198 – 215  263 - 271 | I at position 141 (2)  T at position 141 (1)  Y at position 146 (3)  R at position 149 (1)  F at position 150 (17)  H at position 150 (7)  E at position 153 (3)  I at position 211 (1)  I at position 265 (1)  L at position 268 (1) | R at position 149 (1)  H at position 150 (7)  *c*  *c* | A at position 141 (3)  *c*  *c* | A at position 141 (2)  *c*  *c* |
| Side chains involved in non-covalent interactions between pentamer subunits in the FMDV capsid (Mateo *et al.*, 2003) | **1B**: E96, I99, R103, H106, T108, T111, Q112, L136, T138, Q142, R145, K148, Y183, E193, V197, N199, Q200, F201, M239, K283, Y285, N287, E298, T107    K173  K181  T195  **1C:** K422, R424, M426, H446, H449, E451, D453, L456, N457, I494, T495, K498  D372  Q374 | *c*  H (12), R (5), S (1), A (2), T (5)  D (19), E (5), N (2)  S(25)  *c*  G (2)  T (16), N (3) | *c*  A (2), T (5)  D (5), E (1), N (2)  S (7)  *c*  *c*  T (2) | *c*  *c*  D (26), E (1)  *c*  *c*  E (1)  D (25), F (1) | *c*  *c*  D (9)  *c*  *c*  E (1)  D (8), F (1) |
| H residues identified in the 1B/1C regions and are proposed to mediate 1B/1C hydrogen bonding (Acharya *et al*., 1989). | **1B**: 106  172  230  242  259  **1C**: 388  412  446  449  496 | *c*  *c*  *c*  *c*  *c*  *c*  L (1)  *c*  *c*  *c* | *c*  *c*  *c*  *c*  *c*  *c*  L (1)  *c*  *c*  *c* | *c*  *c*  *c*  *c*  *c*  T (1)  *c*  *c*  *c*  D (1) | *c*  *c*  *c*  *c*  *c*  T (1)  *c*  *c*  *c*  D (1) |
| C residues at these positions are important for the formation a disulphide bond at the base of the G-H loop | **1B:** 215  **1D:** 660 | K (26)  S (15)  N (9)  D (2) | K (8)  S (7)  N (1) | C (27)  S (1) | C (9)  *c* |
| A R residue is important for HS binding (Fry *et al*., 1999). | **1C**: 359 | C (26) | C (8) | H (25)  R (2) | H (8)  R (1) |
| The H residue plays a significant role on FMDV capsid destabilization (v. Vlijmen *et al*., 1998; Ellard *et al*., 1999). | **1C**: 449 | *c* | *c* | *c* | *c* |
| The P residue contributes to the 1A/1B cleavage pocket (Carrillo *et al*., 2005). | **1D**: 715 | *c* | *c* | *c* | *c* |
| The RGD residue is important for receptor binding (Fox *et al.,* 1989; Mason *et al.,* 1994; Berinstein *et al.,* 1995; Jackson *et al*., 1997). | **1D**: 671 - 673 | *c* | *c* | SGD (1) | *c* |
| A L residue is most common and important for virus receptor (αvβ6) recognition and plays a role in the stability of the virus/integrin complex (DiCara *et al*., 2008) | **1D**: 674  677 | M (6)  S (2)  T (3)  H (2)  P(2) | M (1)  *c* | *c*  *c* | *c*  *c* |

***** The aa numbering is according to the P1 alignment in Appendix A8. Note: Not all the sequences referred to in this table is included in Appendix A8. This table is based on the complete alignment of all the isolates included in this study (results not shown).

*c* Refers to the aa residues that are conserved in the P1 alignment.

The numbers in brackets ( ) indicate the number of isolates where there is a difference in aa and the letter next to the brackets refers to the aa change for those isolates.

N The number of isolates aligned
